# Supplementary material for: Conditional Regulation of Gene Expression by Ligand-Induced Occlusion of a MicroRNA Target Sequence
Source: Mol Ther. 2018 Feb 27;26(5):1277–86. doi: 10.1016/j.ymthe.2018.02.021 (PMC5993935; doi:10.1016/j.ymthe.2018.02.021)
Supplement: Document S1. Figures S1 and S2 and Tables S1 and S2 [file mmc1.pdf]

YMTHE, Volume *26*

Supplemental Information

Conditional Regulation of Gene Expression by Ligand-Induced Occlusion of a MicroRNA Target Sequence

Huihui Mou, Guocai Zhong, Matthew R. Gardner, Haimin Wang, Yi-Wen Wang, Dechun Cheng, and Michael Farzan

Fig. S1

A Tet aptamer

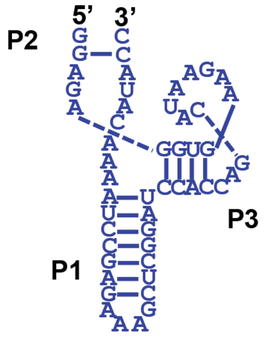

B 3′-type

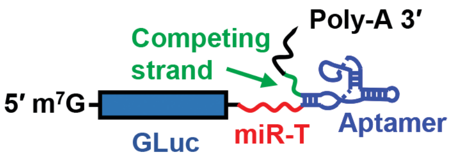

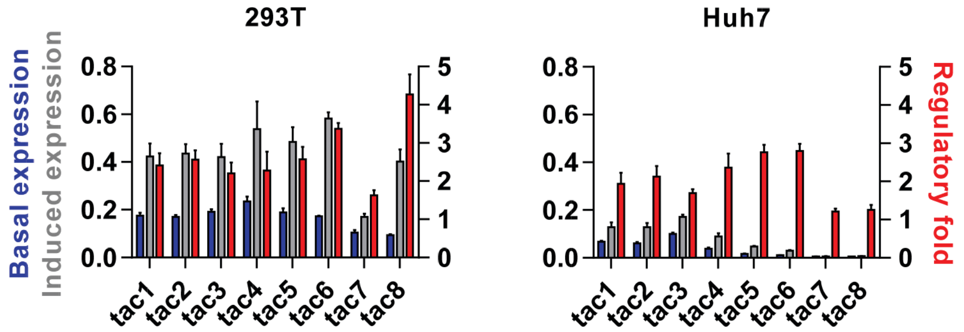

C 5′-type

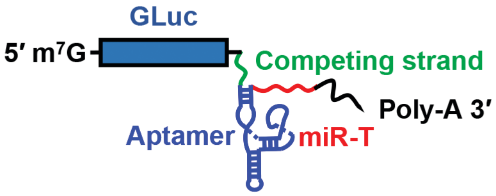

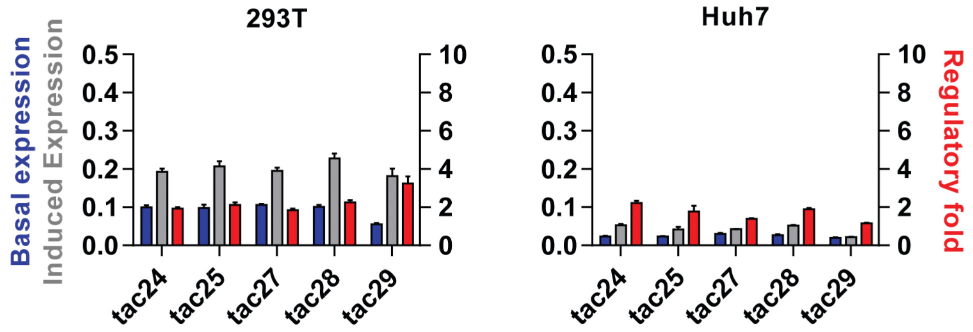

Fig. S2

Mou et al. 2017

A

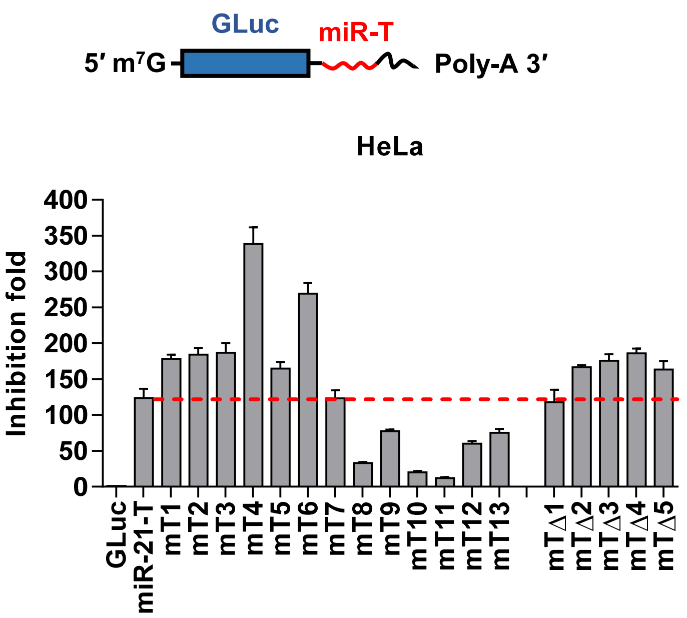

miR-21-T 5′-UCAACAUCAGUCUGAUAAGCUA-3′
miR-21-mT1 5′-UUAACAUCAGUCUGAUAAGCUA-3′
miR-21-mT2 5′-UCGACAUCAGUCUGAUAAGCUA-3′
miR-21-mT3 5′-UCAGCAUCAGUCUGAUAAGCUA-3′
miR-21-mT4 5′-UCAAUAUCAGUCUGAUAAGCUA-3′
miR-21-mT5 5′-UCAACGUCAGUCUGAUAAGCUA-3′
miR-21-mT6 5′-UCAACAUUAGUCUGAUAAGCUA-3′
miR-21-mT7 5′-UCAACAUCGGUCUGAUAAGCUA-3′
miR-21-mT8 5′-UCAACAUCAGUUUGAUAAGCUA-3′
miR-21-mT9 5′-UCAACAUCAGUCUGGUAAGCUA-3′
miR-21-mT10 5′-UCAACAUCAGUCUGAUGAGCUA-3′
miR-21-mT11 5′-UCAACAUCAGUCUGAUAGGCUA-3′
miR-21-mT12 5′-UCAACAUCAGUCUGAUAAGUUA-3′
miR-21-mT13 5′-UCAACAUCAGUCUGAUAAGCUG-3′

miR-21-mTΔ1 5′-CAACAUCAGUCUGAUAAGCUA-3′
miR-21-mTΔ2 5′-AACAUCAGUCUGAUAAGCUA-3′
miR-21-mTΔ3 5′-ACAUCAGUCUGAUAAGCUA-3′
miR-21-mTΔ4 5′-CAUCAGUCUGAUAAGCUA-3′
miR-21-mTΔ5 5′-AUCAGUCUGAUAAGCUA-3′

B

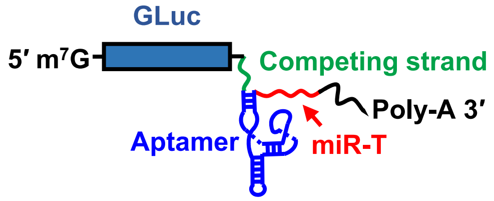

tac29 5′-UCAACAUCAGUCUGAUAAGCUA-3′
tac67 5′-CAACAUCAGUCUGAUAAGCUA-3′
tac68 5′-AACAUCAGUCUGAUAAGCUA-3′
tac69 5′-ACAUCAGUCUGAUAAGCUA-3′
tac70 5′-CAUCAGUCUGAUAAGCUA-3′
tac71 5′-AUCAGUCUGAUAAGCUA-3′

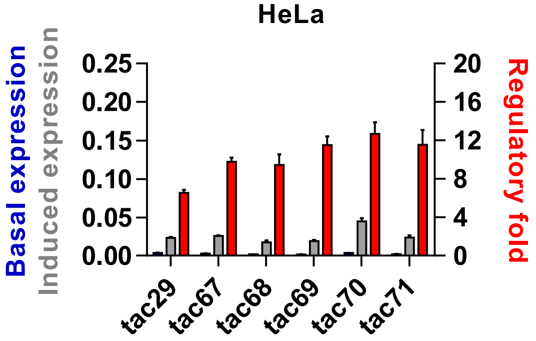

Table S1. List of primers used in this study.

Primers	Sequences
Forward primer	cagtgtggtggaattcaccatgggagtcaaagttctg
Reverse primer-*let-7a*	gccctctagactcgagTGAGGTAGTAGGTTGTATAGTTtttgtttgtttgcgg ccgcttagtcaccaccggcccccttg
Reverse primer-*let-7b*	gccctctagactcgagTGAGGTAGTAGGTTGTGTGGTTtttgtttgtttgcgg ccgcttagtcaccaccggcccccttg
Reverse primer-miR-21	gccctctagactcgagTAGCTTATCAGACTGATGTTGAtttgtttgtttgcgg ccgcttagtcaccaccggcccccttg
Reverse primer-miR-22	gccctctagactcgagAAGCTGCCAGTTGAAGAACTGTtttgtttgtttgcgg ccgcttagtcaccaccggcccccttg
Reverse primer-miR-23	gccctctagactcgagATCACATTGCCAGGGATTTCCtttgtttgtttgcggc cgcttagtcaccaccggcccccttg
Reverse primer-miR-122	gccctctagactcgagTAAGTGTGACAATGGTGTTTGtttgtttgtttgcggc cgcttagtcaccaccggcccccttg

Primer sequences used to introduce miR-T sequences into a *Gaussia* luciferase-reporter plasmids. miR-T sequences are capitalized.

Table S2. On-switch sequences used in this study.

On-switches	Sequences
tac1	UCAACAUCAGUCUGAUAAGCUA-aptamer-UAGCUUAUCA
tac2	UCAACAUCAGUCUGAUAAGCUA-aptamer-UAGCUUAUCAGA
tac3	UCAACAUCAGUCUGAUAAGCUA-aptamer-UAGCUUAUCAGACU
tac4	UCAACAUCAGUCUGAUAAGCUA-aptamer-UGGCUUAUCA
tac5	UCAACAUCAGUCUGAUAAGCUA-aptamer-UGGUUUAUCA
tac6	UCAACAUCAGUCUGAUAAGCUA-aptamer-UGGUUUGUCA
tac7	UCAACAUCAGUCUGAUAAGCUA-aptamer-UGGUUUGUUA

tac8	UCAACAUCAGUCUGAUAAGCUA-aptamer-UGGUUUGUUG
tac9	AACUAUACAACCUACUACCUCA-aptamer-UGAGGUAGUA
tac10	AACUAUACAACCUACUACCUCA-aptamer-UGAGGUAGUAGG
tac11	AACUAUACAACCUACUACCUCA-aptamer-UGAGGUAGUAGGUU
tac12	AACUAUACAACCUACUACCUCA-aptamer-UGGGGUAGUA
tac13	AACUAUACAACCUACUACCUCA-aptamer-UGGGGUGGUA
tac14	AACUAUACAACCUACUACCUCA-aptamer-UGGGGUGGUG
tac15	ACAGUUCUUCAACUGGCAGCUU-aptamer-AAGCUGCCAG
tac16	ACAGUUCUUCAACUGGCAGCUU-aptamer-AAGCUGCCAGUU
tac17	ACAGUUCUUCAACUGGCAGCUU-aptamer-AAGCUGCCAGUUGA
tac18	ACAGUUCUUCAACUGGCAGCUU-aptamer-GAGCUGCCAG
tac19	ACAGUUCUUCAACUGGCAGCUU-aptamer-GGGCUGCCAG
tac20	ACAGUUCUUCAACUGGCAGCUU-aptamer-GGGUUGCCAG
tac21	ACAGUUCUUCAACUGGCAGCUU-aptamer-GGGUUGUCAG
tac22	ACAGUUCUUCAACUGGCAGCUU-aptamer-GGGUUGUUAG
tac23	ACAGUUCUUCAACUGGCAGCUU-aptamer-GGGUUGUUGG
tac24	CUGAUGUUGA-aptamer-UCAACAUCAGUCUGAUAAGCUA
tac25	GACUGAUGUUGA-aptamer-UCAACAUCAGUCUGAUAAGCUA
tac27	CUGAUGUUGG-aptamer-UCAACAUCAGUCUGAUAAGCUA
tac28	CUGGUGUUGG-aptamer-UCAACAUCAGUCUGAUAAGCUA
tac29	UUGGUGUUGG-aptamer-UCAACAUCAGUCUGAUAAGCUA
tac30	UUGUAUAGUU-aptamer-AACUAUACAACCUACUACCUCA
tac31	GGUUGUAUAGUU-aptamer-AACUAUACAACCUACUACCUCA
tac32	UAGGUUGUAUAGUU-aptamer-AACUAUACAACCUACUACCUCA
tac33	UUGUAUGGUU-aptamer-AACUAUACAACCUACUACCUCA

tac34	UUGUGUGGUU-aptamer-AACUAUACAACCUACUACCUCA
tac35	GAAGAACUGU-aptamer-ACAGUUCUUCAACUGGCAGCUU
tac36	UUGAAGAACUGU-aptamer-ACAGUUCUUCAACUGGCAGCUU
tac37	AGUUGAAGAACUGU-aptamer-ACAGUUCUUCAACUGGCAGCUU
tac38	GAAGAAUUGU-aptamer-ACAGUUCUUCAACUGGCAGCUU
tac39	GAAGAGUUGU-aptamer-ACAGUUCUUCAACUGGCAGCUU
tac40	GAAGGGUUGU-aptamer-ACAGUUCUUCAACUGGCAGCUU
tac41	GAGGGGUUGU-aptamer-ACAGUUCUUCAACUGGCAGCUU
tac42	GGGGGGUUGU-aptamer-ACAGUUCUUCAACUGGCAGCUU
tac52	CUGAUGUUGA-aptamer-UCAACAUCAGUCUGAUAAGCUA-aptamer-UAGCUUAUCA
tac53	UUGGUGUUGG-aptamer-UCAACAUCAGUCUGAUAAGCUA-aptamer-UGGUUUGUCA
tac57	UUGGUGUUGG-aptamer-UCAACAUCAGUCUGAUAAGCUA-aptamer-UGGUUUGUC
tac58	UUGGUGUUGG-aptamer-UCAACAUCAGUCUGAUAAGCUA-aptamer-UGGUUUGU
tac59	CGUUUUGGUGUUGG-aptamer-UCAACAUCAGUCUGAUAAGCUA-aptamer-UGGUUUGUCAUUCG
tac60	CGCUUUUGGUGUUGG-aptamer-UCAACAUCAGUCUGAUAAGCUA-aptamer-UGGUUUGUCAUUGCG
tac61	CGCGUUUUGGUGUUGG-aptamer-UCAACAUCAGUCUGAUAAGCUA-aptamer-UGGUUUGUCAUUCGCG
tac62	CGCGCUUUUGGUGUUGG-aptamer-UCAACAUCAGUCUGAUAAGCUA-aptamer-UGGUUUGUCAUUGCGCG
tac63	CGUUUUUGGUGUUGG-aptamer-UCAACAUCAGUCUGAUAAGCUA-aptamer-UGGUUUGUCAUUUCG
tac64	CGCUUUUUGGUGUUGG-aptamer-UCAACAUCAGUCUGAUAAGCUA-aptamer-UGGUUUGUCAUUUGCG

tac65	CGCGUUUUUGGUGUUGG-aptamer-UCAACAUCAGUCUGAUAAGCUA-aptamer-UGGUUUGUCAUUUCGCG
tac66	CGCGCUUUUUGGUGUUGG-aptamer-UCAACAUCAGUCUGAUAAGCUA-aptamer-UGGUUUGUCAUUUGCGCG
tac67	GUUGGUGUUG-aptamer-CAACAUCAGUCUGAUAAGCUA
tac68	GGUUGGUGUU-aptamer-AACAUCAGUCUGAUAAGCUA
tac69	AGGUUGGUGU-aptamer-ACAUCAGUCUGAUAAGCUA
tac70	CAGGUUGGUG-aptamer-CAUCAGUCUGAUAAGCUA
tac71	UCAGGUUGGU-aptamer-AUCAGUCUGAUAAGCUA
tac72	UUGGUGUUGG-aptamer-UCAACAUCAGUCUGAUAAGUUA-aptamer-UGGUUUAUCA
tac73	UUGGUGUUGG-aptamer-UCAACAUCAGUCUGAUGAGCUA-aptamer-UGGUUUAUCA
tac74	CUGGUGUUGG-aptamer-UCAAUAUCAGUCUGAUAAGCUA-aptamer-UGGUUUGUCA
tac75	GUUCGGGGGG-aptamer-UUCUCCGAACGUGUCACGUUU-aptamer-GGGCGUGACA
tac199	CUGGUGUUGG-aptamer-UCAAUAUCAGUCUGAUAAGCUA-aptamer-UGGUUUGUC
tac122	CAAACACCATTGTCACACCCA-aptamer-CGGGGTGTGAAAA
tac209	CGUUUCUGGUGUUGG-aptamer-UCAAUAUCAGUCUGAUAAGCUA-aptamer-UGGUUUGUCUUUCG
tac210	CGCUUUCUGGUGUUGG-aptamer-UCAAUAUCAGUCUGAUAAGCUA-aptamer-UGGUUUGUCUUUGCG

Sequences of the on-switches used in this study. Red indicates miR-T sequence, green indicates competing strands, and cyan indicates transition mismatches. Some dual-type on-switches include a ‘lock-stem’ shown in yellow and separated from the competing strand by a short uracil linker (purple).
