# Supplementary material for: Conditional Regulation of Gene Expression by Ligand-Induced Occlusion of a MicroRNA Target Sequence
Source: Mol Ther. 2018 Feb 27;26(5):1277–86. doi: 10.1016/j.ymthe.2018.02.021 (PMC5993935; doi:10.1016/j.ymthe.2018.02.021)
Supplement: Document S2. Article plus Supplemental Information [file mmc2.pdf]

Molecular Therapy
Original Article

Conditional Regulation of Gene Expression by Ligand-Induced Occlusion of a MicroRNA Target Sequence

Huihui Mou,[1] Guocai Zhong,[1] Matthew R. Gardner,[1] Haimin Wang,[1] Yi-Wen Wang,[1] Dechun Cheng,[1,2] and Michael Farzan[1]

[1]Department of Immunology and Microbiology, The Scripps Research Institute, Jupiter, FL 33458, USA; [2]Department of Parasitology, Harbin Medical University, Harbin 150081, China

RNA switches that modulate gene expression with small molecules have a number of scientific and clinical applications. Here, we describe a novel class of small regulatory on switches based on the ability of a ligand-bound aptamer to promote stem formation between a microRNA target sequence (miR-T) and a complementary competing strand. Two on switch architectures employing this basic concept were evaluated, differing in the location of a tetracycline aptamer and the region of a miR-21 target sequence (miR-21-T) masked by its competing strand. Further optimizations of miR-21-T and its competing strand resulted in tetracycline-regulated on switches that induced luciferase expression by 19-fold in HeLa cells. A similar switch design based on miR-122-T afforded 7-fold regulation when placed in tandem, indicating that this approach can be extended to additional miR-T. Optimized on switches introduced into adeno-associated virus (AAV) vectors afforded 10-fold regulation of two antiviral proteins in AAV-transduced cells. Our data demonstrate that small-molecule-induced occlusion of a miR-T can be used to conditionally regulate gene expression in mammalian cells and suggest that regulatory switches built on this principle can be used to dose expression of an AAV transgene.

INTRODUCTION

Systems that permit external regulation of gene expression can be applied in many scientific and medical contexts. These include construction of synthetic biological networks;[1] regulation of proliferation and protein expression in cells engineered for therapeutic applications, including tumor eradication;[2] and control of expression of biologics delivered by gene therapy vectors.[3] One approach to such regulation has been inspired by natural riboswitches, structured non-coding RNA domains that bind specific metabolites to control gene expression. A riboswitch consists of a small molecular ligand-binding domain (also called aptamer) and an effector domain. Many natural riboswitches utilize ligand-induced base pairing to activate their effector domains, and this mechanism has been successfully utilized to engineer artificial riboswitches functional in yeast or mammalian cells.[4–11] Engineered switches have been used to modulate replication of oncolytic viruses[8] and T cell proliferation[6] and typically use self-cleaving ribozymes for their effector domains. One optimization of this approach, in which an aptamer is used to control a ribozyme, affords approximately 30-fold regulation in cell culture and 5-fold regulation *in vivo*.[11] Another approach relevant here uses aptamers fused to an short hairpin RNA (shRNA) element, allowing a small molecule to regulate Dicer-mediated cleavage, thereby activating expression of the shRNA target gene.[12,13] However, the dynamic ranges of these switches have been relatively narrow, limiting their utility. Alternative effector domains may provide more versatile regulation with wider regulatory ranges, ultimately enabling more practical uses of these switches.

In this study, we describe an efficient class of on switches that utilize a cell's endogenous microRNA-mediated silencing machinery to regulate gene expression. RNA silencing requires an RNA-induced silencing complex (RISC), which recognizes its target mRNA through base pairing interactions between a RISC-associated microRNA and its microRNA target sequence (miR-T).[14] As target site accessibility determines the efficiency of RISC-mediated target recognition and silencing,[15] we hypothesized that base pairing induced by a ligand-bound aptamer would limit accessibility of the miR-T, prevent silencing, and enable target gene expression (Figure 1). Here, we prove this principle and demonstrate one application of these on switches, namely regulation of a gene therapy transgene.

RESULTS

Two Types of MicroRNA Target-Based On Switches

The silencing efficiency of a microRNA limits the maximum efficiency of the miR-T-based on switches. We thus selected target sequences of five ubiquitously expressed microRNAs (*let-7a*, *let-7b*, miR-21, miR-22, and miR-23; Figure 2A)[16–18] and determined, in

Received 20 October 2017; accepted 22 February 2018;
https://doi.org/10.1016/j.ymthe.2018.02.021.

Correspondence: Guocai Zhong, Department of Immunology and Microbiology, The Scripps Research Institute, 130 Scripps Way, Jupiter, FL 33458, USA.
E-mail: gzhong@scripps.edu
Correspondence: Michael Farzan, Department of Immunology and Microbiology, The Scripps Research Institute, 130 Scripps Way, Jupiter, FL 33458, USA.
E-mail: mfarzan@scripps.edu

This is an open access article under the CC BY license (http://creativecommons.org/licenses/by/4.0/).

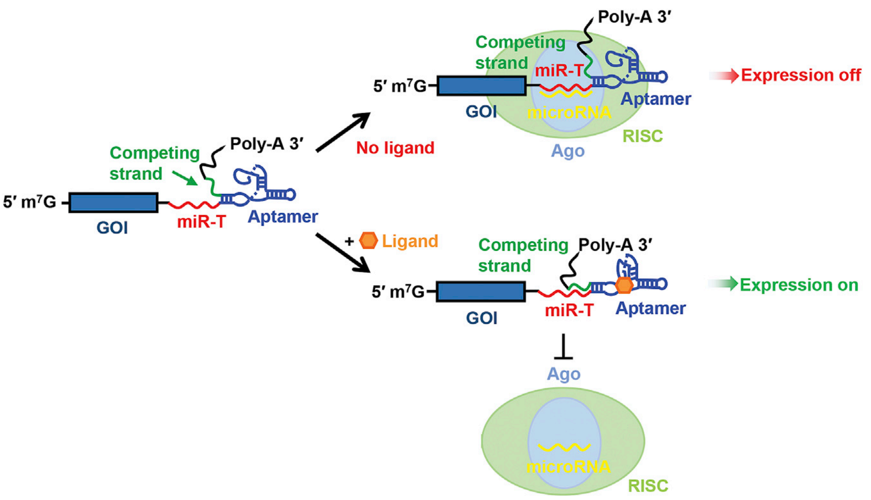

Figure 1. Representation of a MicroRNA-Target-Based On Switch

We designed a series of on switches based on the ability of a ligand-bound aptamer to promote occlusion of a microRNA target sequence (miR-T). In the absence of ligand, the miR-T (red), included at the 3′ UTR of a gene-of-interest (GOI) mRNA, is exposed to the RNA-induced silencing complex (RISC) (light green), and translation of the GOI is suppressed. The aptamer ligand (orange) induces a conformational change in the aptamer (blue), promoting the formation of a stem composed of a competing strand (green) and part of the miR-T. Because this region of the miR-T is occluded, the RISC complex cannot access the miR-T and the GOI is expressed.

three cell lines, their respective efficiencies in mediating silencing of a *Gaussia* luciferase (GLuc) reporter gene. Introduction of the miR-21 target sequence (miR-21-T) into the 3′ UTR of the GLuc reporter gene consistently resulted in more efficient silencing activity. We therefore used miR-21-T for most of our subsequent studies. To test whether aptamer-promoted stem formation could be used to occlude a miR-T and inhibit microRNA-induced silencing, we introduced a tetracycline (Tet)-binding aptamer (Figure S1A) and a competing strand adjacent to the miR-21-T. This Tet aptamer[19,20] has been shown to bind Tet with high affinity[21] and undergo significant conformational changes in its presence.[22] Two basic designs were investigated. In the first case, diagrammed in Figure 2B, the aptamer (blue) and a miR-T competing strand (green) were placed at the 3′ of miR-21-T (red). In this 3′-type design, the Tet-bound aptamer stabilizes an extended stem, including the 3′ region of miR-21-T and the competing strand. In the second case, the aptamer and its competing strand were placed at the 5′ of the miR-21-T (5′-type; Figure 2C).

We first sought to optimize the competing strands of 3′-type on switches. 3′-type on switches with competing strands of various lengths, or bearing increasing numbers of mismatches, were investigated by measuring basal GLuc expression in the absence of Tet (blue bars), induced expression in the presence of Tet (gray bars), and by calculating fold increase due to Tet (red bars). We observed that a competing strand of 10 nt and three transition mismatches, present in on switch tac6, consistently exhibited the highest dynamic range of this class in the three cell lines tested (Figures 2B and S1B), approximately 3-fold in HeLa cells. A similar pattern was observed with 5′-type on switches (Figures 2C and S1C). Specifically, the on switch tac29, with a 10-nt competing strand and three transition mismatches, again exhibited the widest dynamic range, approximately 6-fold in HeLa cells. We also characterized 3′- and 5′-type on switches with *let-7a* and miR-22 target sequences (not shown). In general, the dynamic ranges of these switches were lower due to the lower silencing activities of their respective microRNAs. We conclude that the length of the competing strand, the number of mismatches, and the overall microRNA activity within the cells determine the efficiency of miR-T-based on switches. To validate the mechanism by which these on switches control gene expression, we examined the ability of Tet to regulate association of a soluble form of tac29 to bind bead-bound miR-21. We observed that Tet could significantly limit binding of tac29 to miR-21 in this cell-free system (Figure 2D) and infer that it does so by promoting the occlusion of the miR-21-T region of tac29.

Optimizations of Combined 3′- and 5′-type On Switches

We then sought to combine 3′ and 5′ types into dual-type on switches (Figure 3A). The dual-type on switch tac52 combines tac1 and tac24, without any competing-strand mismatches. On switch tac53 combines tac6 and tac29, among the most efficient 3′- and 5′-type on switches, respectively. We also explored variants of tac53, whose 3′ competing strand was shortened by one (tac57) or two (tac58) nucleotides. On switch tac52 provided a lower dynamic range than its tac1 and tac24 constituents, likely because the miR-21-T was largely occluded, even in the absence of Tet. However, both tac53 and tac57 outperformed their tac6 and tac29 constituents, exhibiting 12-fold and 13-fold dynamic ranges in HeLa cells, respectively. We conclude that dual-type on switches can provide wider dynamic ranges than switches composed of a single aptamer and competing strand.

We hypothesized that introduction of complementary sequences at the base of the 3′ and 5′ competing strands might stabilize a conformation induced by aptamer binding (Figure 3B). We therefore tested tac53 variants bearing one of four complementary "lock-stem" sequences (yellow), separated by two- or three-uracil linkers. Four such variants—tac60, tac63, tac64, and tac65—consistently exhibited modestly wider dynamic ranges than tac53, suggesting that, in the presence of Tet, these lock-stem sequences helped stabilize a conformation in which miR-21-T is occluded. We also explored the impact of single-transition mutations in miR-21-T on the efficiency of miR-21 silencing. We observed that some transitions modulated the efficiency of silencing (Figure S2A), and we accordingly introduced

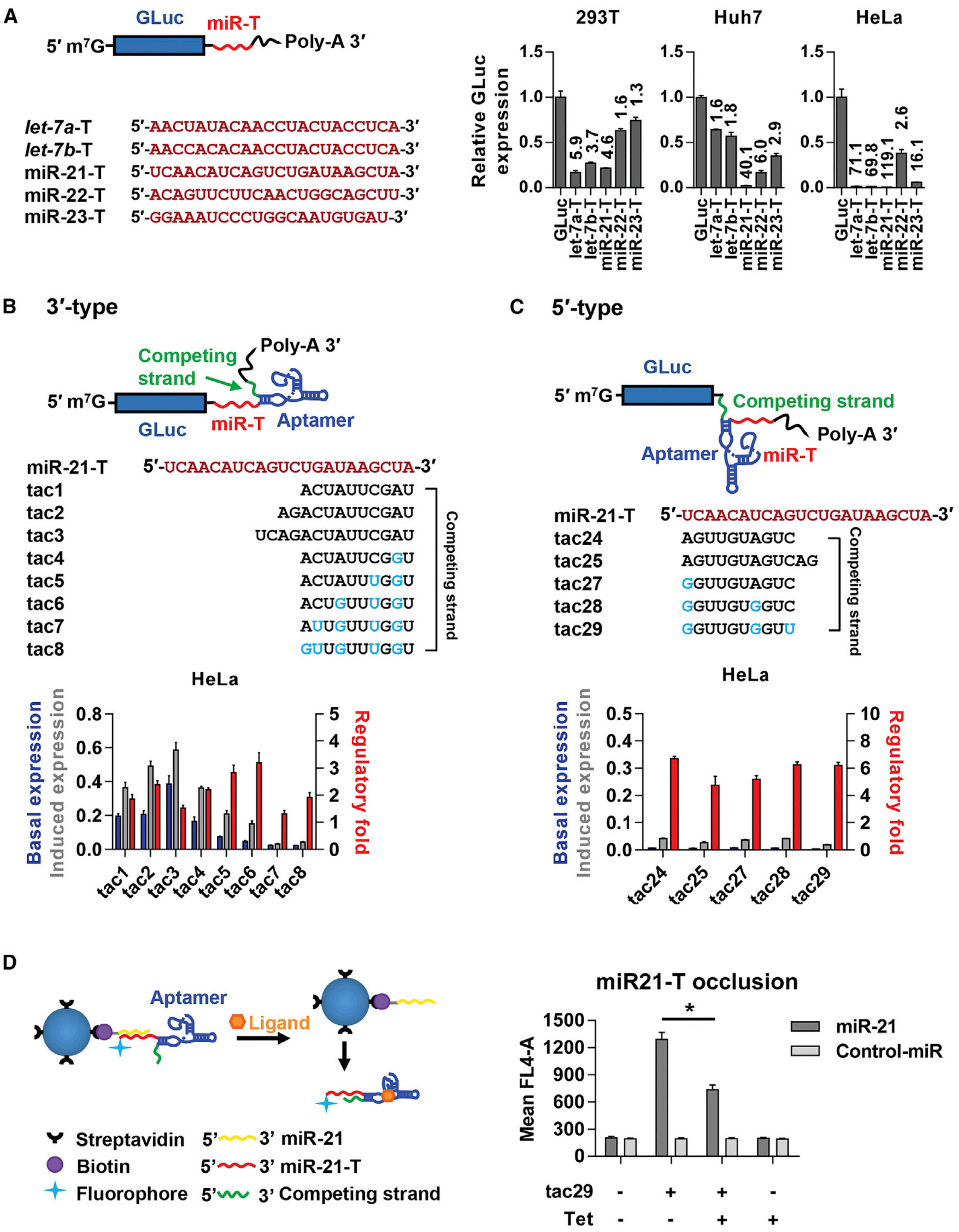

Figure 2. Characterization of Two On Switch Designs

(A) miR-Ts of five microRNAs reported to be ubiquitous were introduced into the 3′ UTRs of a *Gaussia* luciferase (GLuc) reporter gene, as shown. miR-T sequences are provided at the left. Plasmids expressing these GLuc genes were co-transfected into 293T, Huh7, or HeLa cells with a plasmid expressing *Cypridina* luciferase (CLuc), used as a transfection control. Bars indicate GLuc expression normalized to GLuc expression without a miR-T, and numbers above the bars indicate fold inhibition, again relative to the GLuc-only control. (B) A representation of a 3′-type on switch is shown, in which the ligand-bound aptamer induces stem formation between the 3′ region of the miR-T and a competing strand. The miR-21 target sequence (miR-21-T) is shown in red, with competing-strand sequences of individual switches shown underneath. Light blue indicates a mismatch between the miR-21-T and the competing strand. HeLa cells were transfected with plasmids encoding *Gaussia* luciferase (GLuc) bearing the indicated

(legend continued on next page)

some of these transitions into miR-21-T of tac53 (Figure 3C). We observed that transitions that increased the efficiency of silencing also increased the dynamic range of the corresponding tac53-variant tac74, whereas those transitions that impaired silencing resulted in decreased dynamic ranges (e.g., tac72 and tac73). We also investigated the impact of deletions of miR-21-T by comparing the 5′-type on switch tac29 with tac29 variants bearing progressively shorter miR-21-T (Figure S2B). We observed that tac70, a tac29 variant with an 18-nt miR-21-T, exhibited a greater than 12-fold dynamic range, the maximum dynamic range of any 3′- or 5′-type switches. We conclude that alteration of the miR-T can improve the dynamic ranges of these on switches.

We then sought to combine features of the dual-type on switches exhibiting the widest dynamic ranges. We observed that a modified switch tac199, which is derived from tac57 and includes the transition mismatch present in the target sequence of tac74, exhibited a wider dynamic range than either original on switch (Figure 3D). Further, when we introduced the lock stems of tac63 and tac64 into tac199, generating tac209 and 210, we observed an additional increase in dynamic range to 19-fold (Figure 3E). We then compared the five dual-type and the two 5′-type on switches with the widest dynamic ranges in a single assay, confirming that the dual-type switch tac210 provided the widest dynamic range of the on switches constructed here (Figure 3F).

Further Improvement and Generalization of miR-T-Based Regulation

We then explored the ability of exogenously expressed miR-21 to improve the narrow regulatory range of tac210 in 293T cells, which afforded less efficient regulation of miR-21-T-based on switches (Figures 2A, S1B, and S1C). Expression of one, two, or three primary miR-21 sequences expanded the regulatory range to 6-fold, indicating that low level of miR-21 limits the range of tac210 in 293T cells (Figure 4A). Thus, this class of on switches could be adapted to cells lacking a particular microRNA by expressing that microRNA exogenously. To assess the generality of these switches, we developed a 3′-type switch, tac122, using the target region of miR-122. This microRNA is primarily expressed in hepatic cells, and we accordingly used the hepatic cell line Huh7. A miR-122 target site (miR-122-T) limited expression of a GLuc reporter gene by 23-fold (Figure 4B), substantially less than that observed using miR-21-T in HeLa cells (119-fold; Figure 2A). Accordingly, tac122 afforded only 4-fold regulation in Huh7, but regulation could be doubled by placing additional tac122 sequences in tandem (Figure 4C). These data show that these on switch designs can be generalized to other microRNA target sequences and cell types and identify two ways of increasing the regulatory efficiency of these switches. Specifically, one can increase expression of the relevant microRNA, and one can place these on switches in tandem.

On Switch Regulation of Adeno-associated Virus Transgenes

We finally explored the ability of tac199 and tac210 to regulate expression of a transgene delivered by an adeno-associated virus (AAV) vector. The on switch tac199 afforded 14-fold regulation of GLuc in AAV-transduced HeLa cells (Figure 5A) and afforded 9-fold regulation of the HIV-1 entry inhibitor CD4-Ig in the same setting (Figure 5B). The on switch tac210 regulated AAV-mediated expression of CD4-Ig and the HIV-1-neutralizing antibody VRC01 11-fold and 7-fold, respectively (Figures 5C and 5D). Thus, the on switches described here can be used to regulate the expression of AAV transgenes.

DISCUSSION

The RNAi pathway has been exploited both scientifically and clinically, most notably through the use of shRNA and microRNA to suppress expression of cellular and viral genes.[23,24] Here, we describe a novel class of RNA switches that conditionally regulate gene expression in mammalian cells through occlusion of a miR-T (Figures 1 and 2D). We assessed a number of designs using this basic idea and identified switches that provided up to 19-fold regulation of gene expression in HeLa cells. We observed that the dynamic ranges of these switches are dependent on the efficiency of RISC-mediated silencing (Figures 2A, 2B, S2B, and S2C). In addition, they depend on free access to the miR-T in the absence of Tet (affecting basal expression) and on efficient masking of the miR-T in its presence (affecting Tet-induced expression). Thus, the annealing energy of the competing strand has an optimum between two extremes, which we approximated in this system with a 10-nt competing strand bearing three transition mismatches with its miR-T (Figures 2B and 2C). We observed that combinations of two such switches, simultaneously occluding the 5′ and 3′ regions of the miR-T, could be more efficient than any single switch (Figure 3A). We also observed better regulation by locking the ends of the annealing strands with short complementary sequences (lock stems; Figure 3B). Interestingly, the efficiency of silencing could be improved by single transitions in the miR-21-T (Figure S2A), an observation used to further improve the regulatory range of these switches (tac74 in Figure 3C).

on switch at its 3′ UTR. GLuc expression was compared in the presence and absence of 100 μM tetracycline (Tet). Blue bars indicate GLuc expression in the absence of Tet ("basal expression"), and gray bars indicate GLuc expression in the presence of Tet ("induced expression"). Values on left vertical axis indicate percent GLuc expression relative to that of a control GLuc plasmid lacking any regulatory elements. Red bars indicate the fold upregulation of expression induced by Tet ("regulatory fold"). (C) An experiment is shown similar to that shown in (B) except that the aptamer and the competing strand are placed at 5′ of the miR-21-T, and a 5′ region of miR-21-T is occluded by ligand-induced stem formation, as diagramed. (D) Magnetic beads conjugated to streptavidin were bound to a biotinylated single-stranded DNA encoding mature miR-21. In the absence of Tet, a soluble switch sequence based on tac29 associated with this miR-21 sequence is shown. Tet inhibited specifically inhibits this association, indicating the miR-21-T region is partially occluded after Tet association. Asterisk indicates at p value of 0.0113 (Student's two-tailed t test). Data points represent a mean ± SD of three replicates for (A)–(C) and two for (D). Each experiment was performed at least twice with similar results.

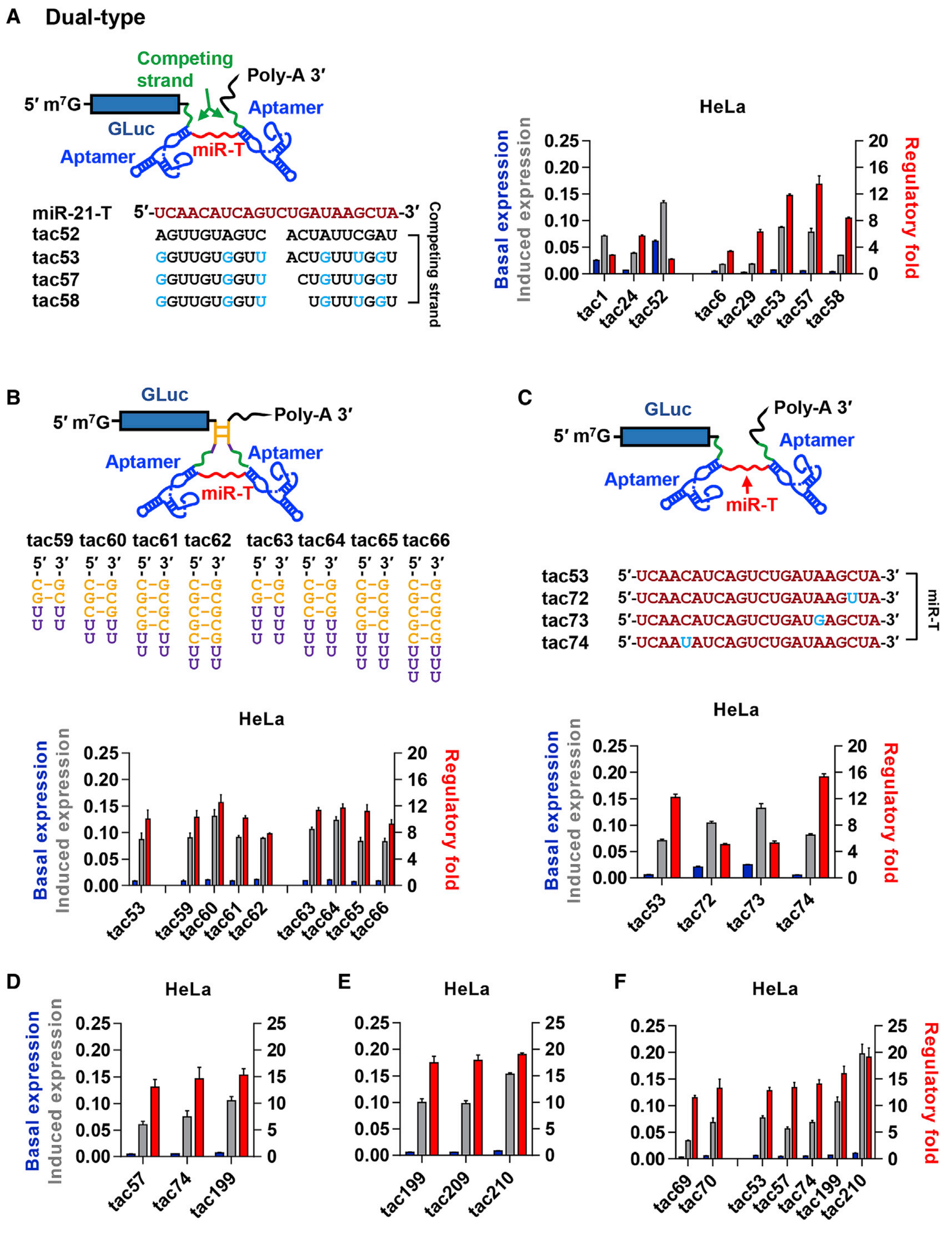

Figure 3. Optimization of On Switches

(A) An experiment similar to those in Figure 2B except that 5′-type and 3′-type on switches are combined to generate dual-type on switch variants. (B) An experiment is shown similar to those in Figure 2B except that a complementary "lock stem" (yellow), followed by a 2- or 3-uracil linker, was placed adjacent to the competing strands in the dual-type on switch tac53. (C) An experiment is shown similar to those in Figure 2B except that the miR-21-T region of the dual-type on switch tac53 was modified with single transitions (blue). (D) The dual-type on switch tac199, modified from tac57 so that its miR-21-T region is that of tac74, is compared with tac57 and tac74. (E) The dual-type on switches tac209 and tac210 are based on tac199, modified to include the lock stem regions of tac63 and tac64, respectively, and are compared with tac199. (F) A direct comparison of on switches with the greatest dynamic ranges among 5′-type and dual-type on switches is shown. Data points in all studies represent a mean ± SD of three replicates and were performed at least twice with similar results.

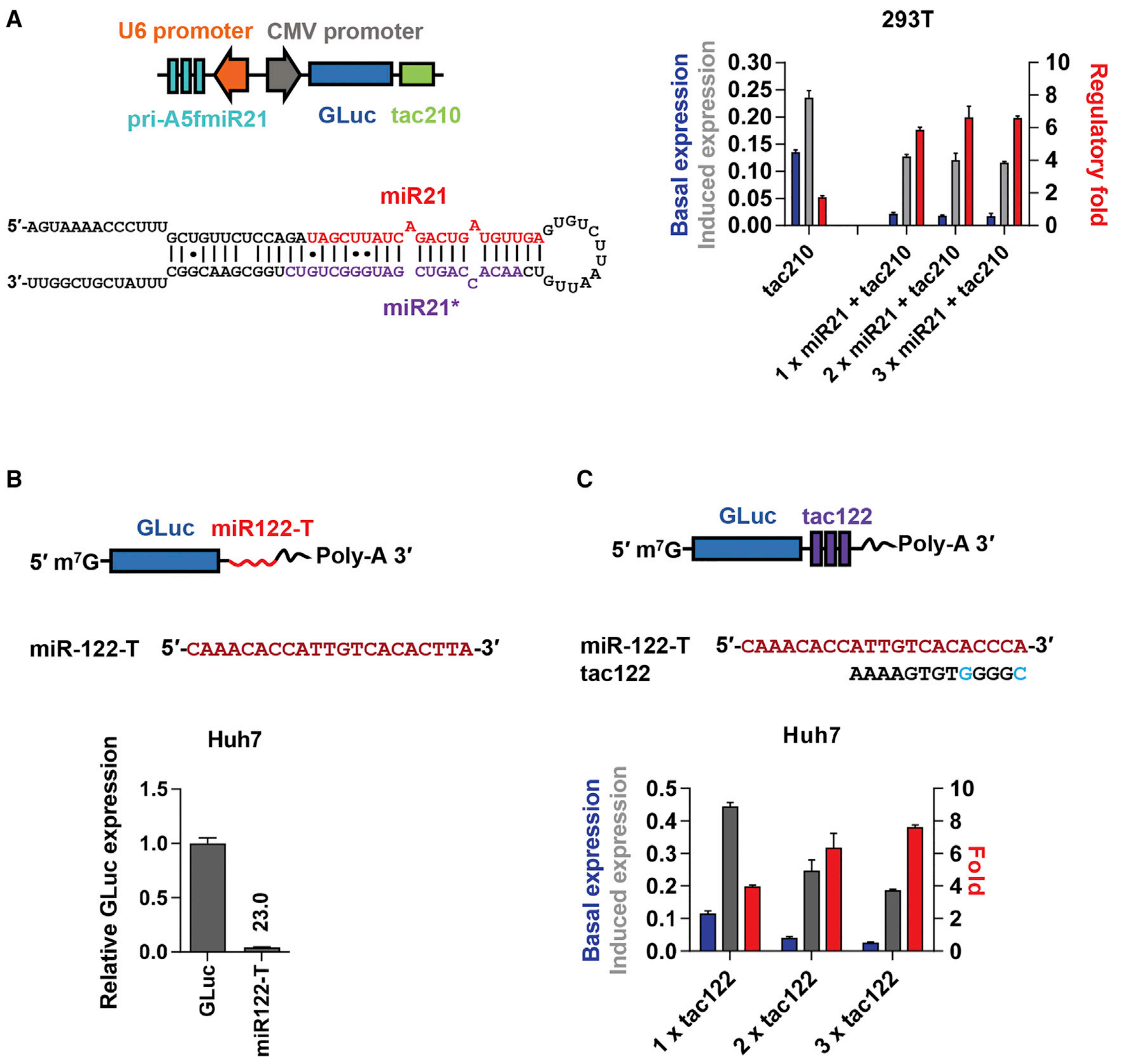

Figure 4. Further Improvement and Generalization of miR-T-Based Regulation

(A) On switch regulation can be improved with exogenous miR-21. One, two, or three artificial primary miR-21 constructs, driven by a U6 promoter, were included in plasmid expressing GLuc regulated by the dual-type on switch tac210. Plasmids, including primary miR-21 sequences or tac210 alone, were transfected into 293T cells, and luciferase activity was measured as in Figure 2B. (B) An experiment similar to those in Figure 2A is shown, except that the efficiency of miR-122-T was characterized in Huh7 cells. (C) An experiment is shown similar to those in Figure 2B, except that miR-122-T was used as a target sequence and 1, 2, or 3 tandem 3′-type switches were characterized. Data points in all studies represent a mean ± SD of three replicates and were performed at least twice with similar results.

The miR-T-based switches we described here have several properties that make them very useful for conditional control of gene expression in scientific and medical applications. First, they are relatively small, typically no more than a few hundred nucleotides, and can therefore be easily introduced into vectors, such as those based on AAV, with strict packaging limits.[25] Second, unlike Tet-On systems, they do not require expression of an exogenous foreign protein that is likely to elicit cell-based immunity to transgene-expressing cells.[26] Finally, they are programmable. They can be tailored to many aptamer-ligand pairs and to diverse miR-T, including those responsive to exogenously expressed, non-native microRNAs (Figures 4A and 4B).

There are several approaches that might further increase the utility of these on switches. For example, we observed that more efficient silencing activity leads to wider dynamic ranges. Thus, HeLa cells, with high levels of miR-21 activity, exhibited greater regulatory ranges than did 293T or Huh7 cells, each with less efficient miR-21 activities (Figures 2B, 2C, S1B, and S1C). Moreover, when miR-21 was introduced into 293T cells exogenously, the dynamic range of these on switches increased (Figure 4A). This observation suggests that exogenous expression of microRNAs can be used to increase the efficiency of this system as well as make its activity less cell type dependent. Our system might be further improved by simultaneously controlling expression of these

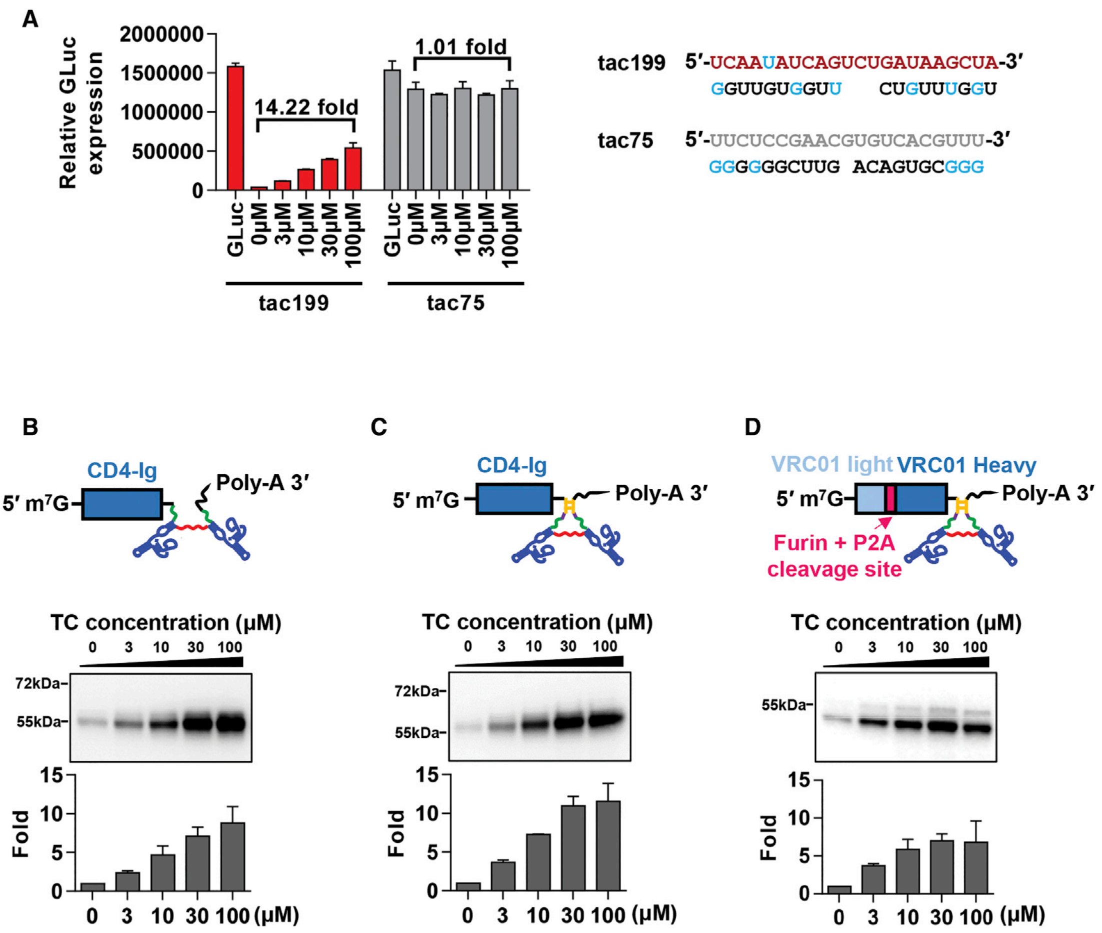

Figure 5. Dual-type On Switches Regulate the Expression of AAV-Expressed Transgenes

(A) Tac199 and the dual-type control construct tac75 bearing a control sequence in the place of the miR-21-T and its complementary competing strands were used to regulate a GLuc reporter gene in AAV-transduced HeLa cells and analyzed as in Figure 2B. (B and C) Tac199 (B) or tac210 (C) were incorporated into the 3′ UTR of an expression cassette for the HIV-1 entry inhibitor CD4-Ig. (D) Tac210 was also introduced into 3′ UTR of the expression cassette of the HIV-1-neutralizing antibody VRC01, bearing VRC01 heavy and light chains separated by a furin cleavage site and the P2A ribosomal skip sequence. Adeno-associated virus (AAV) vectors bearing these cassettes were produced in 293T cells, and filtered viral stocks were used to transduce HeLa cells in the presence of the indicated Tet concentrations. Proteins were precipitated by Protein-A Sepharose beads and analyzed by western blot, and band intensities were quantified by Quantity One 1-D software. Data are presented as a mean ± SD of two independent wells from the same experiment and are representative of at least two independent experiments with similar results.

exogenous microRNAs, using one of several reported RNA-based systems for doing so.[5,12,13,27] We also observed that our best switches induced only 20% of luciferase activity observed in the absence of a regulatory switch (Figures 3D and 3E), suggesting that the RISC can outcompete the competing strand for access to the miR-T. It is possible that an aptamer ligand with better access to the nucleus could promote masking of the miR-T before it encounters the cytoplasmic RISC. Finally, Tet is not an ideal aptamer ligand in other respects as well. Its bioavailability is poor in some tissues, including muscle, a relatively safe target tissue used in many gene therapy applications. Moreover, its long-term use is associated with several unwanted side effects. Given the number of switches that now use aptamers, the development of aptamers to more bioavailable, better tolerated small molecules is clearly warranted.

In summary, we have described a new class of efficient on switches and shown that these switches can regulate expression of proteins delivered by AAV vectors. We therefore propose that, with further optimizations, these switches can be used to regulate long-term expression of therapeutically useful biologics and perhaps in other scientific and clinical settings.

MATERIALS AND METHODS

Cell Lines and Reagents

HEK293T (ATCC CRL-3216, VA, USA), HeLa (ATCC CCL-2), and Huh7 cells (Life Technologies, Thermo Fisher Scientific, Carlsbad, CA, USA) were maintained in growth media composed of DMEM (Life Technologies) supplemented with 2 mM glutamine (Life Technologies), 1% non-essential amino acids (Life Technologies), 100 U/mL penicillin and 100 μg/mL streptomycin (Life Technologies), and

10% FBS (Sigma-Aldrich, St. Louis, MI, USA) at 37°C in 5% CO_2. To assess the effect of Tet treatment on on-switch activity, medium was changed to growth medium containing 10% Tet-free FBS (Takara Bio USA, Mountain View, CA, USA), to which varying concentrations of Tet were added. 40 mM HEPES (Life Technologies) was supplemented to the medium to provide extra buffering capacity. Tet solution was prepared by dissolving the Tet hydrochloride (Sigma-Aldrich) into water (Sigma-Aldrich) to the final concentration 20 mM. This solution was centrifuged at 3,000 × *g* at room temperature for 30 min, and the bottom 1 mL was discarded after centrifugation. *Gaussia* luciferase (GLuc) assay buffer consists of 7.5 mM sodium acetate, 250 mM sodium sulfate, 250 mM sodium chloride, and 4 μM coelenterazine native (Biosynth Chemistry and Biology, Itasca, IL, USA) at pH 5.0.

Plasmids

GLuc-reporter plasmids in which miR-T sequences were included at their 3′ UTRs were generated by PCR amplification of a GLuc gene with the reverse primers, including miR-T sequences (Table S1). Inserts for creation of GLuc constructs bearing miR-T on switches were synthesized by Integrated DNA Technologies (IDT, Coralville, IA, USA). Sequences of switches are shown in Table S2. Ligation was performed using In-Fusion HD Cloning Kit (Takara Bio USA) according to manufacturer's instructions. Constructs combining GLuc bearing on switch tac210 with a cassette for primary miR-21 (pri-miR-21) production were generated by inversely inserting the synthesized U6 promoter and 1–3 copies of pri-A5fmiR-21 into the BglII site of the GLuc-tac210 plasmid. Pri-A5fmiR-21 comprises miR-21 and miR-21* inserted into the frame of a previously described artificial pri-microRNA.[28] Construct and pri-A5fmiR-21 sequences are illustrated in Figure 3F. A plasmid expressing *Cypridina* luciferase (CLuc), used in these studies as the transfection control, was purchased from New England Biolabs (NEB) (Ipswich, MA, USA). AAV-vector plasmids expressing HIV entry inhibitors, including miR-T on switches, were constructed from the pAAV-MCS plasmid (Agilent Technologies, Santa Clara, CA, USA).

Transient Transfection and Luciferase Assays

To compare the inhibition effect of miR-T introduced into the 3′ UTR region of the GLuc reporter, cells seeded in 96-well plates were co-transfected with 10 ng of plasmids encoding GLuc with or without miR-T sequences, together with 5 ng of plasmid expressing CLuc, using 0.5 μL Lipofectamine 2000 (Thermo Fisher Scientific) according to manufacturer's instructions. After a 4-hr incubation, medium of transfected cells was changed to growth medium containing 10% FBS. Medium was refreshed 24 hr post-transfection, and supernatant was harvested at 48 hr post-transfection for measurement of luciferase activity. To investigate the regulatory effect of miR-T on switches, cells seeded in 96-well plates were co-transfected with 10 ng plasmids encoding GLuc, with or without miR-T on switches, and 5 ng plasmid expressing CLuc using 0.5 μL Lipofectamine 2000, according to manufacturer's instructions. After a 4-hr incubation, medium of transfected cells was changed to growth medium containing 10% Tet-free FBS. Three hours later, transfected cells were cultured with Tet-free medium supplemented with 0, 3, 10, 30, or 100 μM Tet. Medium was refreshed at 24 hr post-transfection, and the supernatant was harvested at 48 hr post-transfection for measurement of luciferase activity. To measure GLuc luciferase activity, 20 μL of cell culture supernatant from each sample was transferred to a black opaque assay plate (Corning, New York, USA), and then 100 μL/well of GLuc assay buffer was added by the injector of the multi-label plate reader (PerkinElmer, USA). To measure CLuc activity, the Cypridina Luciferase Flash Assay Kit (Pierce, Thermo Fisher Scientific) was used, and the measurement was performed according to manufacturer's instructions. Briefly, 20 μL of cell culture supernatant from each sample was transferred to a black opaque assay plate (Corning), and then 50 μL/well of CLuc assay buffer was added by the injector of the multi-label plate reader (PerkinElmer, Waltham, MA, USA). Statistical analyses were performed using GraphPad Prism 6.0, and data were presented as mean ± SD.

In Vitro Occlusion of miR-T by Competing Strands

In this experiment, the wash steps and coupling of biotinylated DNA to streptavidin beads were performed according to the manufacturer's instructions. Briefly, the beads (Dynabeads MyOne Streptavidin C1, Life Technologies) in the vial were resuspended, and desired volume was transferred to a tube and washed 3× with equal volumes of binding and washing (B&W) buffer (5 mM Tris-HCl [pH 7.5], 0.5 mM EDTA, and 1 M NaCl). For RNA applications, beads were washed twice with equal volume of Solution A (diethylpyrocarbonate [DEPC]-treated 0.1 M NaOH and DEPC-treated 0.05 M NaCl) followed by one more wash with equal volume of Solution B (DEPC-treated 0.i M NaCl). Washed beads were then suspended in equal volume of 2× B&W buffer (10 mM Tris-HCl [pH 7.5], 1 mM EDTA, and 2 M NaCl) and aliquoted for different coupling applications. Synthesized 5′-biotin-modified miR-21 or control-miR DNA sequences (10 mM in DEPC-H_2O) were added to prepared beads and incubated at 300 rpm, 25°C for 15 min on ThermoMixer (Eppendorf, Hamburg, Germany). After coupling, the biotinylated oligo-coated beads were separated magnetically for 2 or 3 min and washed 3× with B&W buffer as well as one more wash with equal volume of TNaK buffer (20 mM Tris-HCl [pH 7.5], 5 mM KCl, and 140 mM NaCl).[29] Beads were then suspended in 2× volume of TNaK buffer. 40 μL beads from previous step was aliquoted to each tube and suspended with equal volume of strand replacement buffer (TNaK buffer supplemented with 1 μM 22-nt-randomized DNA oligo, including the same percentage of four bases with miR-21-T, IDT, 5 mM $MgCl_2$, as well as 0.5 μL RNase inhibitor). The tac29 RNA oligo with 5′-ATTO 608N fluorophore modification was synthesized by IDT. The delivered tac29 was resuspended in DEPC-H_2O, and 40 nM was added to the prepared beads from previous step with or without the presence of 100 μM Tet. The reactions were covered and incubated at 300 rpm, 25°C for 2 hr on ThermoMixer. After the incubation, beads in each tube were washed 3× with 100 μL TNaK buffer and suspended in 100 μL TNaK buffer for flow analysis, and fluorophore signal was analyzed with Accuri (BD Biosciences, Franklin Lakes, NJ, USA).

AAV Production and Transduction

AAV vectors carrying various expression cassette and on switch variants were produced using AAV Helper-Free System (Agilent Technologies) according to manufacturer's instructions. Briefly, 70%–80% confluent HEK293T cells in 100-mm plate were co-transfected with 10 μg pAAV vector encoding GLuc, HIV entry inhibitors CD4-Ig, or VRC01 with on switches, 10 μg pAAV-RC/1, and 10 μg pHelper using the CalPhos Transfection Kit (Takara Bio USA). VRC01 includes both heavy- and light-chain genes separated by sequence encoding the porcine teschovirus-1 2A (P2A) peptide that promotes ribosomal skipping and a furin cleavage site to remove the P2A peptide.[30] After a 6 hr incubation at 37°C, medium was removed and replaced with 10 mL fresh growth medium. After another 48 hr incubation at 37°C, AAV vector was harvested by collecting cell supernatant filtered with a 0.45-μm filter (Millipore, Billerica, MA, USA) to eliminate cell debris. To transduce cells with AAV vectors, HeLa cells were seeded in 12-well plates one day prior to transduction to achieve 80%–90% confluence at transduction. Cells were transduced by adding 1 mL clarified virus stock and centrifuging at 1,250 × *g* at 25°C for 45 min, followed by incubating at 37°C for another 3 hr 15 min. After transduction, medium of transduced cells was changed to growth medium containing 10% Tet-free FBS. After another 3-hr incubation, transduced cells were cultured with Tet-free medium supplemented with 0, 3, 10, 30, or 100 μM Tet. Medium was refreshed at 24 hr post-transduction, and the supernatant was harvested at 48 hr post-transduction for luciferase measurement or western blot analysis.

Immunoprecipitation and Western Blot Analysis

Supernatants of HeLa cells expressing the HIV-1 entry inhibitor CD4-Ig or the HIV-1-neutralizing antibody VRC01 were harvested and clarified by centrifuging at 3,000 × *g*, 4°C for 5 min. Clarified supernatant was transferred to a new tube and incubated with 20 μL of 50% (V/V) Protein-A Sepharose beads (GE Healthcare, Chicago, IL, USA) at 4°C overnight. After two washes with ice-cooled PBS without calcium and magnesium, beads were heated to 95°C in 2× reducing Laemmli sample buffer and eluant was analyzed by 10% SDS-PAGE (Novex 10% Tris-Glycine Mini Gels, Invitrogen, Thermo Fisher Scientific). Proteins were transferred to a polyvinylidene fluoride membrane (Invitrolon PVDF/Filter Paper, Invitrogen), which was then blocked with PBS containing 5% fetal calf serum (FCS) and 0.05% Tween 20. Proteins were visualized by horseradish peroxidase (HRP)-conjugated goat-anti-human-immunoglobulin G (IgG) (Jackson ImmunoResearch Laboratories, West Grove, PA, USA). Bands on the blots were imaged with an ImageQuant LAS 4000 Mini imager (GE Healthcare), and their intensities were quantified by Quantity One 1-D software (Bio-Rad, Hercules, CA, USA). Statistical analysis was performed with GraphPad Prism 6.0, and data were presented as mean ± SD.

Statistical Analysis

All analyses were performed using GraphPad Prism 6.0 software, and the significance of *in vitro* miR-21-T occlusion assay was assessed by Student's two-tailed t test.

SUPPLEMENTAL INFORMATION

Supplemental Information includes two figures and two tables and can be found with this article online at https://doi.org/10.1016/j.ymthe.2018.02.021.

AUTHOR CONTRIBUTIONS

H.M., G.Z., and M.F. designed the research. H.M., G.Z., M.R.G., H.W., Y.-W.W., and D.C. performed experiments and analyzed data. H.M., G.Z., and M.F. wrote the paper. All authors reviewed the paper.

CONFLICTS OF INTEREST

The authors declare no conflict of interest.

ACKNOWLEDGMENTS

We thank Dr. Hyeryun Choe for her insightful comments and careful reading of the manuscript. This work is supported by NIH grants R37 AI091476, P01 AI100263, DP1 DA043912, and R44 AI124832 (to M.F.).

REFERENCES

1. Win, M.N., and Smolke, C.D. (2008). Higher-order cellular information processing with synthetic RNA devices. Science *322*, 456–460.
2. Straathof, K.C., Pulè, M.A., Yotnda, P., Dotti, G., Vanin, E.F., Brenner, M.K., Heslop, H.E., Spencer, D.M., and Rooney, C.M. (2005). An inducible caspase 9 safety switch for T-cell therapy. Blood *105*, 4247–4254.
3. Seiwert, T.Y., Darga, T., Haraf, D., Blair, E.A., Stenson, K., Cohen, E.E., Salama, J.K., Villaflor, V., Witt, M.E., Lingen, M.W., et al. (2013). A phase I dose escalation study of Ad GV.EGR.TNF.11D (TNFerade™ Biologic) with concurrent chemoradiotherapy in patients with recurrent head and neck cancer undergoing reirradiation. Ann. Oncol. *24*, 769–776.
4. Win, M.N., and Smolke, C.D. (2007). A modular and extensible RNA-based gene-regulatory platform for engineering cellular function. Proc. Natl. Acad. Sci. USA *104*, 14283–14288.
5. Kumar, D., An, C.I., and Yokobayashi, Y. (2009). Conditional RNA interference mediated by allosteric ribozyme. J. Am. Chem. Soc. *131*, 13906–13907.
6. Chen, Y.Y., Jensen, M.C., and Smolke, C.D. (2010). Genetic control of mammalian T-cell proliferation with synthetic RNA regulatory systems. Proc. Natl. Acad. Sci. USA *107*, 8531–8536.
7. Beilstein, K., Wittmann, A., Grez, M., and Suess, B. (2015). Conditional control of mammalian gene expression by tetracycline-dependent hammerhead ribozymes. ACS Synth. Biol. *4*, 526–534.
8. Ketzer, P., Kaufmann, J.K., Engelhardt, S., Bossow, S., von Kalle, C., Hartig, J.S., Ungerechts, G., and Nettelbeck, D.M. (2014). Artificial riboswitches for gene expression and replication control of DNA and RNA viruses. Proc. Natl. Acad. Sci. USA *111*, E554–E562.
9. Klauser, B., Atanasov, J., Siewert, L.K., and Hartig, J.S. (2015). Ribozyme-based aminoglycoside switches of gene expression engineered by genetic selection in S. cerevisiae. ACS Synth. Biol. *4*, 516–525.
10. Townshend, B., Kennedy, A.B., Xiang, J.S., and Smolke, C.D. (2015). High-throughput cellular RNA device engineering. Nat. Methods *12*, 989–994.
11. Zhong, G., Wang, H., Bailey, C.C., Gao, G., and Farzan, M. (2016). Rational design of aptazyme riboswitches for efficient control of gene expression in mammalian cells. eLife *5*, e18858.
12. An, C.I., Trinh, V.B., and Yokobayashi, Y. (2006). Artificial control of gene expression in mammalian cells by modulating RNA interference through aptamer-small molecule interaction. RNA *12*, 710–716.

13. Beisel, C.L., Bayer, T.S., Hoff, K.G., and Smolke, C.D. (2008). Model-guided design of ligand-regulated RNAi for programmable control of gene expression. Mol. Syst. Biol. *4*, 224.

14. Wilson, R.C., and Doudna, J.A. (2013). Molecular mechanisms of RNA interference. Annu. Rev. Biophys. *42*, 217–239.

15. Ameres, S.L., Martinez, J., and Schroeder, R. (2007). Molecular basis for target RNA recognition and cleavage by human RISC. Cell *130*, 101–112.

16. Tang, X., Gal, J., Zhuang, X., Wang, W., Zhu, H., and Tang, G. (2007). A simple array platform for microRNA analysis and its application in mouse tissues. RNA *13*, 1803–1822.

17. Lagos-Quintana, M., Rauhut, R., Meyer, J., Borkhardt, A., and Tuschl, T. (2003). New microRNAs from mouse and human. RNA *9*, 175–179.

18. Lagos-Quintana, M., Rauhut, R., Lendeckel, W., and Tuschl, T. (2001). Identification of novel genes coding for small expressed RNAs. Science *294*, 853–858.

19. Berens, C., Thain, A., and Schroeder, R. (2001). A tetracycline-binding RNA aptamer. Bioorg. Med. Chem. *9*, 2549–2556.

20. Xiao, H., Edwards, T.E., and Ferré-D'Amaré, A.R. (2008). Structural basis for specific, high-affinity tetracycline binding by an in vitro evolved aptamer and artificial riboswitch. Chem. Biol. *15*, 1125–1137.

21. Müller, M., Weigand, J.E., Weichenrieder, O., and Suess, B. (2006). Thermodynamic characterization of an engineered tetracycline-binding riboswitch. Nucleic Acids Res. *34*, 2607–2617.

22. Förster, U., Weigand, J.E., Trojanowski, P., Suess, B., and Wachtveitl, J. (2012). Conformational dynamics of the tetracycline-binding aptamer. Nucleic Acids Res. *40*, 1807–1817.

23. Davidson, B.L., and McCray, P.B., Jr. (2011). Current prospects for RNA interference-based therapies. Nat. Rev. Genet. *12*, 329–340.

24. Ipsaro, J.J., and Joshua-Tor, L. (2015). From guide to target: molecular insights into eukaryotic RNA-interference machinery. Nat. Struct. Mol. Biol. *22*, 20–28.

25. Sheridan, C. (2011). Gene therapy finds its niche. Nat. Biotechnol. *29*, 121–128.

26. Favre, D., Blouin, V., Provost, N., Spisek, R., Porrot, F., Bohl, D., Marmé, F., Chérel, Y., Salvetti, A., Hurtrel, B., et al. (2002). Lack of an immune response against the tetracycline-dependent transactivator correlates with long-term doxycycline-regulated transgene expression in nonhuman primates after intramuscular injection of recombinant adeno-associated virus. J. Virol. *76*, 11605–11611.

27. Tuleuova, N., An, C.I., Ramanculov, E., Revzin, A., and Yokobayashi, Y. (2008). Modulating endogenous gene expression of mammalian cells via RNA-small molecule interaction. Biochem. Biophys. Res. Commun. *376*, 169–173.

28. Fang, W., and Bartel, D.P. (2015). The menu of features that define primary microRNAs and enable de novo design of microRNA genes. Mol. Cell *60*, 131–145.

29. Martini, L., Ellington, A.D., and Mansy, S.S. (2016). An in vitro selection for small molecule induced switching RNA molecules. Methods *106*, 51–57.

30. Kim, J.H., Lee, S.R., Li, L.H., Park, H.J., Park, J.H., Lee, K.Y., Kim, M.K., Shin, B.A., and Choi, S.Y. (2011). High cleavage efficiency of a 2A peptide derived from porcine teschovirus-1 in human cell lines, zebrafish and mice. PLoS ONE *6*, e18556.

YMTHE, Volume *26*

Supplemental Information

Conditional Regulation of Gene Expression by Ligand-Induced Occlusion of a MicroRNA Target Sequence

Huihui Mou, Guocai Zhong, Matthew R. Gardner, Haimin Wang, Yi-Wen Wang, Dechun Cheng, and Michael Farzan

Fig. S1

A Tet aptamer

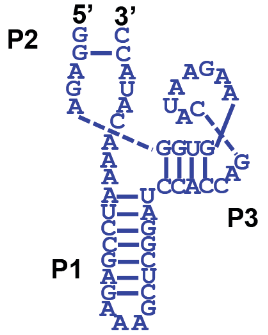

B 3′-type

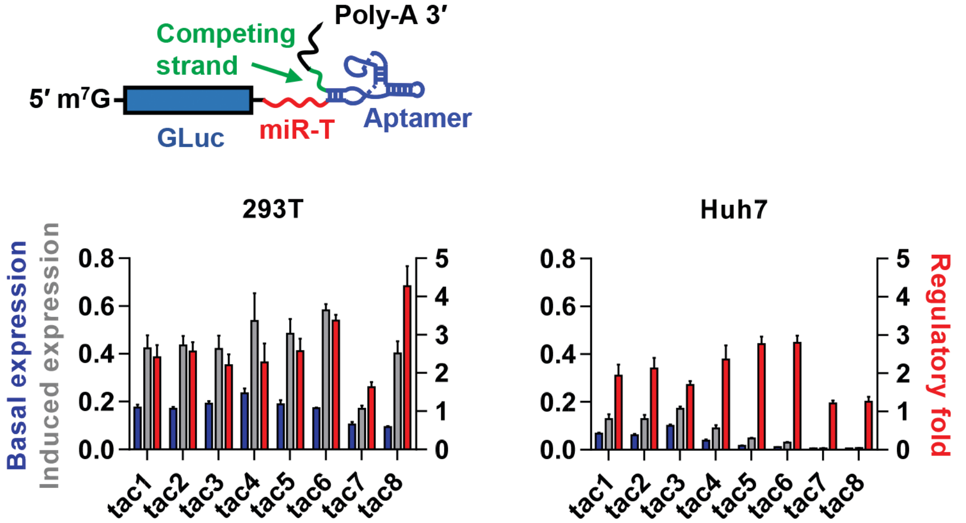

C 5′-type

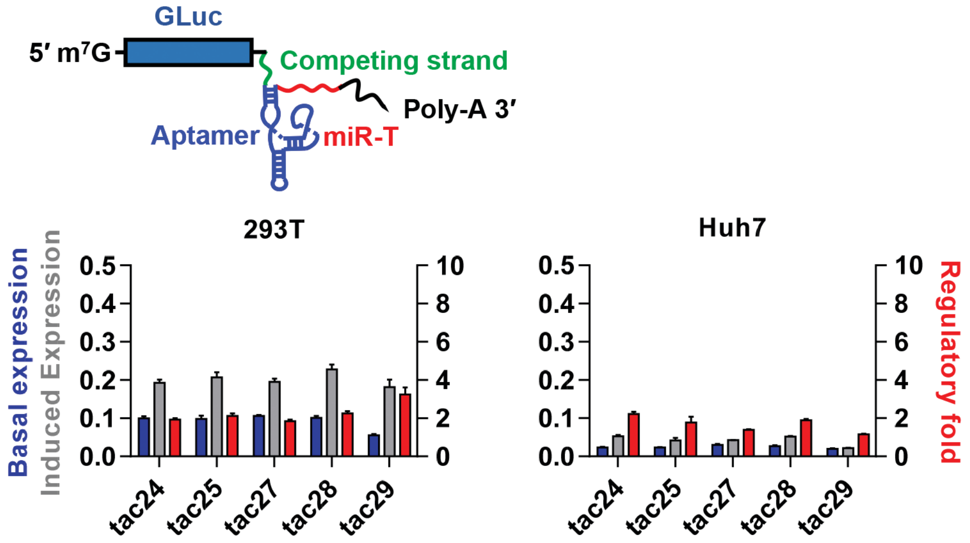

Fig. S2

Mou et al. 2017

A

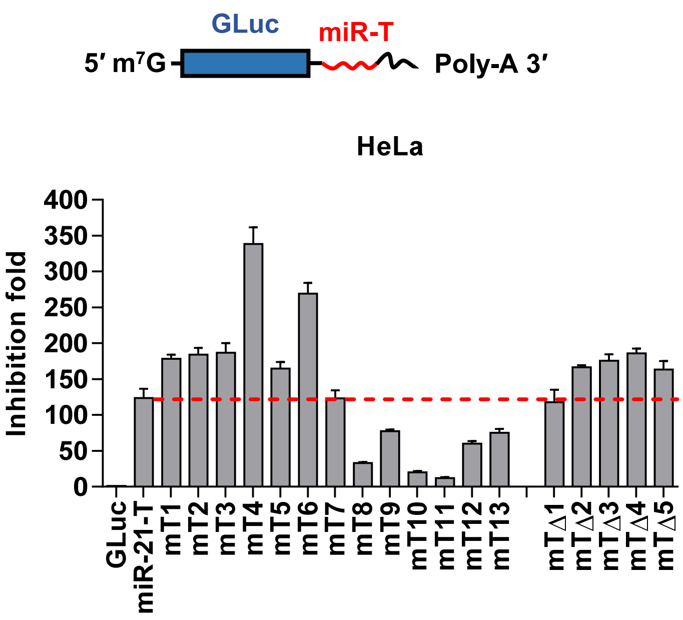

miR-21-T	5′-UCAACAUCAGUCUGAUAAGCUA-3′
miR-21-mT1	5′-UUAACAUCAGUCUGAUAAGCUA-3′
miR-21-mT2	5′-UCGACAUCAGUCUGAUAAGCUA-3′
miR-21-mT3	5′-UCAGCAUCAGUCUGAUAAGCUA-3′
miR-21-mT4	5′-UCAAUAUCAGUCUGAUAAGCUA-3′
miR-21-mT5	5′-UCAACGUCAGUCUGAUAAGCUA-3′
miR-21-mT6	5′-UCAACAUUAGUCUGAUAAGCUA-3′
miR-21-mT7	5′-UCAACAUCGGUCUGAUAAGCUA-3′
miR-21-mT8	5′-UCAACAUCAGUUUGAUAAGCUA-3′
miR-21-mT9	5′-UCAACAUCAGUCUGGUAAGCUA-3′
miR-21-mT10	5′-UCAACAUCAGUCUGAUGAGCUA-3′
miR-21-mT11	5′-UCAACAUCAGUCUGAUAGGCUA-3′
miR-21-mT12	5′-UCAACAUCAGUCUGAUAAGUUA-3′
miR-21-mT13	5′-UCAACAUCAGUCUGAUAAGCUG-3′
miR-21-mTΔ1	5′-CAACAUCAGUCUGAUAAGCUA-3′
miR-21-mTΔ2	5′-AACAUCAGUCUGAUAAGCUA-3′
miR-21-mTΔ3	5′-ACAUCAGUCUGAUAAGCUA-3′
miR-21-mTΔ4	5′-CAUCAGUCUGAUAAGCUA-3′
miR-21-mTΔ5	5′-AUCAGUCUGAUAAGCUA-3′

B

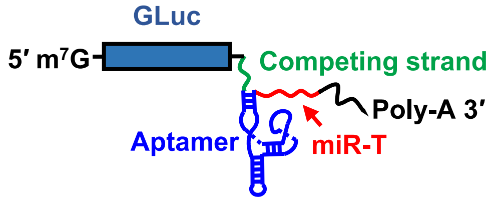

tac29	5′-UCAACAUCAGUCUGAUAAGCUA-3′
tac67	5′-CAACAUCAGUCUGAUAAGCUA-3′
tac68	5′-AACAUCAGUCUGAUAAGCUA-3′
tac69	5′-ACAUCAGUCUGAUAAGCUA-3′
tac70	5′-CAUCAGUCUGAUAAGCUA-3′
tac71	5′-AUCAGUCUGAUAAGCUA-3′

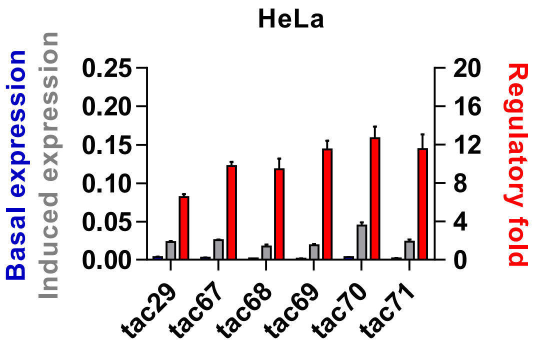

Table S1. List of primers used in this study.

Primers	Sequences
Forward primer	cagtgtggtggaattcaccatgggagtcaaagttctg
Reverse primer-*let-7a*	gccctctagactcgagTGAGGTAGTAGGTTGTATAGTTtttgtttgtttgcgg ccgcttagtcaccaccggcccccttg
Reverse primer-*let-7b*	gccctctagactcgagTGAGGTAGTAGGTTGTGTGGTTtttgtttgtttgcgg ccgcttagtcaccaccggcccccttg
Reverse primer-miR-21	gccctctagactcgagTAGCTTATCAGACTGATGTTGAtttgtttgtttgcgg ccgcttagtcaccaccggcccccttg
Reverse primer-miR-22	gccctctagactcgagAAGCTGCCAGTTGAAGAACTGTtttgtttgtttgcgg ccgcttagtcaccaccggcccccttg
Reverse primer-miR-23	gccctctagactcgagATCACATTGCCAGGGATTTCCtttgtttgtttgcggc cgcttagtcaccaccggcccccttg
Reverse primer-miR-122	gccctctagactcgagTAAGTGTGACAATGGTGTTTGtttgtttgtttgcggc cgcttagtcaccaccggcccccttg

Primer sequences used to introduce miR-T sequences into a *Gaussia* luciferase-reporter plasmids. miR-T sequences are capitalized.

Table S2. On-switch sequences used in this study.

On-switches	Sequences
tac1	**UCAACAUCAGUCUGAUAAGCUA-aptamer-UAGCUUAUCA**
tac2	**UCAACAUCAGUCUGAUAAGCUA-aptamer-UAGCUUAUCAGA**
tac3	**UCAACAUCAGUCUGAUAAGCUA-aptamer-UAGCUUAUCAGACU**
tac4	**UCAACAUCAGUCUGAUAAGCUA-aptamer-UGGCUUAUCA**
tac5	**UCAACAUCAGUCUGAUAAGCUA-aptamer-UGGUUUAUCA**
tac6	**UCAACAUCAGUCUGAUAAGCUA-aptamer-UGGUUUGUCA**
tac7	**UCAACAUCAGUCUGAUAAGCUA-aptamer-UGGUUUGUUA**

tac8	UCAACAUCAGUCUGAUAAGCUA-aptamer-UGGUUUGUUG
tac9	AACUAUACAACCUACUACCUCA-aptamer-UGAGGUAGUA
tac10	AACUAUACAACCUACUACCUCA-aptamer-UGAGGUAGUAGG
tac11	AACUAUACAACCUACUACCUCA-aptamer-UGAGGUAGUAGGUU
tac12	AACUAUACAACCUACUACCUCA-aptamer-UGGGGUAGUA
tac13	AACUAUACAACCUACUACCUCA-aptamer-UGGGGUGGUA
tac14	AACUAUACAACCUACUACCUCA-aptamer-UGGGGUGGUG
tac15	ACAGUUCUUCAACUGGCAGCUU-aptamer-AAGCUGCCAG
tac16	ACAGUUCUUCAACUGGCAGCUU-aptamer-AAGCUGCCAGUU
tac17	ACAGUUCUUCAACUGGCAGCUU-aptamer-AAGCUGCCAGUUGA
tac18	ACAGUUCUUCAACUGGCAGCUU-aptamer-GAGCUGCCAG
tac19	ACAGUUCUUCAACUGGCAGCUU-aptamer-GGGCUGCCAG
tac20	ACAGUUCUUCAACUGGCAGCUU-aptamer-GGGUUGCCAG
tac21	ACAGUUCUUCAACUGGCAGCUU-aptamer-GGGUUGUCAG
tac22	ACAGUUCUUCAACUGGCAGCUU-aptamer-GGGUUGUUAG
tac23	ACAGUUCUUCAACUGGCAGCUU-aptamer-GGGUUGUUGG
tac24	CUGAUGUUGA-aptamer-UCAACAUCAGUCUGAUAAGCUA
tac25	GACUGAUGUUGA-aptamer-UCAACAUCAGUCUGAUAAGCUA
tac27	CUGAUGUUGG-aptamer-UCAACAUCAGUCUGAUAAGCUA
tac28	CUGGUGUUGG-aptamer-UCAACAUCAGUCUGAUAAGCUA
tac29	UUGGUGUUGG-aptamer-UCAACAUCAGUCUGAUAAGCUA
tac30	UUGUAUAGUU-aptamer-AACUAUACAACCUACUACCUCA
tac31	GGUUGUAUAGUU-aptamer-AACUAUACAACCUACUACCUCA
tac32	UAGGUUGUAUAGUU-aptamer-AACUAUACAACCUACUACCUCA
tac33	UUGUAUGGUU-aptamer-AACUAUACAACCUACUACCUCA

tac34	UUGUGUGGUU-aptamer-AACUAUACAACCUACUACCUCA
tac35	GAAGAACUGU-aptamer-ACAGUUCUUCAACUGGCAGCUU
tac36	UUGAAGAACUGU-aptamer-ACAGUUCUUCAACUGGCAGCUU
tac37	AGUUGAAGAACUGU-aptamer-ACAGUUCUUCAACUGGCAGCUU
tac38	GAAGAAUUGU-aptamer-ACAGUUCUUCAACUGGCAGCUU
tac39	GAAGAGUUGU-aptamer-ACAGUUCUUCAACUGGCAGCUU
tac40	GAAGGGUUGU-aptamer-ACAGUUCUUCAACUGGCAGCUU
tac41	GAGGGGUUGU-aptamer-ACAGUUCUUCAACUGGCAGCUU
tac42	GGGGGGUUGU-aptamer-ACAGUUCUUCAACUGGCAGCUU
tac52	CUGAUGUUGA-aptamer-UCAACAUCAGUCUGAUAAGCUA-aptamer-UAGCUUAUCA
tac53	UUGGUGUUGG-aptamer-UCAACAUCAGUCUGAUAAGCUA-aptamer-UGGUUUGUCA
tac57	UUGGUGUUGG-aptamer-UCAACAUCAGUCUGAUAAGCUA-aptamer-UGGUUUGUC
tac58	UUGGUGUUGG-aptamer-UCAACAUCAGUCUGAUAAGCUA-aptamer-UGGUUUGU
tac59	CGUUUUGGUGUUGG-aptamer-UCAACAUCAGUCUGAUAAGCUA-aptamer-UGGUUUGUCAUUCG
tac60	CGCUUUUGGUGUUGG-aptamer-UCAACAUCAGUCUGAUAAGCUA-aptamer-UGGUUUGUCAUUGCG
tac61	CGCGUUUUGGUGUUGG-aptamer-UCAACAUCAGUCUGAUAAGCUA-aptamer-UGGUUUGUCAUUCGCG
tac62	CGCGCUUUUGGUGUUGG-aptamer-UCAACAUCAGUCUGAUAAGCUA-aptamer-UGGUUUGUCAUUGCGCG
tac63	CGUUUUUGGUGUUGG-aptamer-UCAACAUCAGUCUGAUAAGCUA-aptamer-UGGUUUGUCAUUUCG
tac64	CGCUUUUUGGUGUUGG-aptamer-UCAACAUCAGUCUGAUAAGCUA-aptamer-UGGUUUGUCAUUUGCG

tac65	CGCGUUUUUGGUGUUGG-aptamer-UCAACAUCAGUCUGAUAAGCUA-aptamer-UGGUUUGUCAUUUCGCG
tac66	CGCGCUUUUUGGUGUUGG-aptamer-UCAACAUCAGUCUGAUAAGCUA-aptamer-UGGUUUGUCAUUUGCGCG
tac67	GUUGGUGUUG-aptamer-CAACAUCAGUCUGAUAAGCUA
tac68	GGUUGGUGUU-aptamer-AACAUCAGUCUGAUAAGCUA
tac69	AGGUUGGUGU-aptamer-ACAUCAGUCUGAUAAGCUA
tac70	CAGGUUGGUG-aptamer-CAUCAGUCUGAUAAGCUA
tac71	UCAGGUUGGU-aptamer-AUCAGUCUGAUAAGCUA
tac72	UUGGUGUUGG-aptamer-UCAACAUCAGUCUGAUAAGUUA-aptamer-UGGUUUAUCA
tac73	UUGGUGUUGG-aptamer-UCAACAUCAGUCUGAUGAGCUA-aptamer-UGGUUUAUCA
tac74	CUGGUGUUGG-aptamer-UCAAUAUCAGUCUGAUAAGCUA-aptamer-UGGUUUGUCA
tac75	GUUCGGGGGG-aptamer-UUCUCCGAACGUGUCACGUUU-aptamer-GGGCGUGACA
tac199	CUGGUGUUGG-aptamer-UCAAUAUCAGUCUGAUAAGCUA-aptamer-UGGUUUGUC
tac122	CAAACACCATTGTCACACCCA-aptamer-CGGGGTGTGAAAA
tac209	CGUUUCUGGUGUUGG-aptamer-UCAAUAUCAGUCUGAUAAGCUA-aptamer-UGGUUUGUCUUUCG
tac210	CGCUUUCUGGUGUUGG-aptamer-UCAAUAUCAGUCUGAUAAGCUA-aptamer-UGGUUUGUCUUUGCG

Sequences of the on-switches used in this study. Red indicates miR-T sequence, green indicates competing strands, and cyan indicates transition mismatches. Some dual-type on-switches include a 'lock-stem' shown in yellow and separated from the competing strand by a short uracil linker (purple).
